# The Role of Sodium Valproate in Clozapine-Associated Cardiotoxicities: The Importance of Cellular and Systemic Energetics

**DOI:** 10.1007/s12012-026-10204-w

**Published:** 2026-10-16

**Authors:** Amanda Groenewald, Amelia S. Power, Kathryn E. Burns, Malcolm D. Tingle, Marie-Louise Ward

**Affiliations:** 1 Department of Physiology, Faculty of Medical and Health Sciences, University of Auckland, Auckland, New Zealand; 2 Department of Pharmacology and Clinical Pharmacology, Faculty of Medical and Health Sciences, The University of Auckland, Auckland, New Zealand

**Keywords:** Clozapine, Valproate, Energetics, Mitochondria, Metabolism, Cardiotoxicity

## Abstract

The antipsychotic drug clozapine is associated with one of the broadest spectra of cardiac adverse effects. Many of these are of unknown aetiology, hindering clinicians’ ability to prevent, diagnose, and treat them. Risk factors for clozapine-associated cardiotoxicities include concomitant valproic acid therapy and comorbidities associated with the dysregulation of systemic metabolism, such as obesity and diabetes. Therefore, this review aims to summarise existing evidence for the adverse effects of these drugs on cellular and systemic energetics. Both drugs are strongly associated with mitochondrial dysfunction and oxidative stress, but in different tissues. Clozapine may be bioactivated within cardiomyocytes and their mitochondria, producing reactive species that damage these organelles, thus impairing oxidative phosphorylation. Valproate may have similar effects in hepatocytes but doesn’t appear to be bioactivated in all cell types, though it reportedly has modest inhibitory effects on fatty acid oxidation. Both drugs affect substrate supply, altering serum lipid, carbohydrate, and ketone levels. In concert, these medications are likely to impair cardiac metabolic flexibility and energetics. Considering the absolute dependence of cardiomyocyte function on continuous energy supply, the dysregulation of cardiomyocyte energetics is likely to have a significant role in the development of clozapine-associated cardiotoxicities. However, limited investigation has been made of the effects of these drugs in cardiomyocytes specifically. Rectifying this could inform improved prevention, detection, and management of cardiotoxicities to facilitate the development of clinical strategies to enable the safer use of these medicines.

**Graphical Abstract:**

Overview of the potential effects of clozapine and valproic acid on cardiomyocyte energetics. Highlighted regions are those where clozapine (orange) or valproic acid (purple) have been shown to have some effect, with colour-coded arrows to indicate the direction of the effect. Partially shaded regions indicate those areas where contradictory effects have been reported in different experimental models. Key enzymes, indicated in brackets, include glutamine synthetase (GS), glutamate synthase (GLS), glutamate dehydrogenase (GDH), glutamate-pyruvate transaminase (GPT), lactate dehydrogenase (LDH), pyruvate dehydrogenase (PDH), and fatty acyl-CoA synthetase (FACS).

**Figure:**
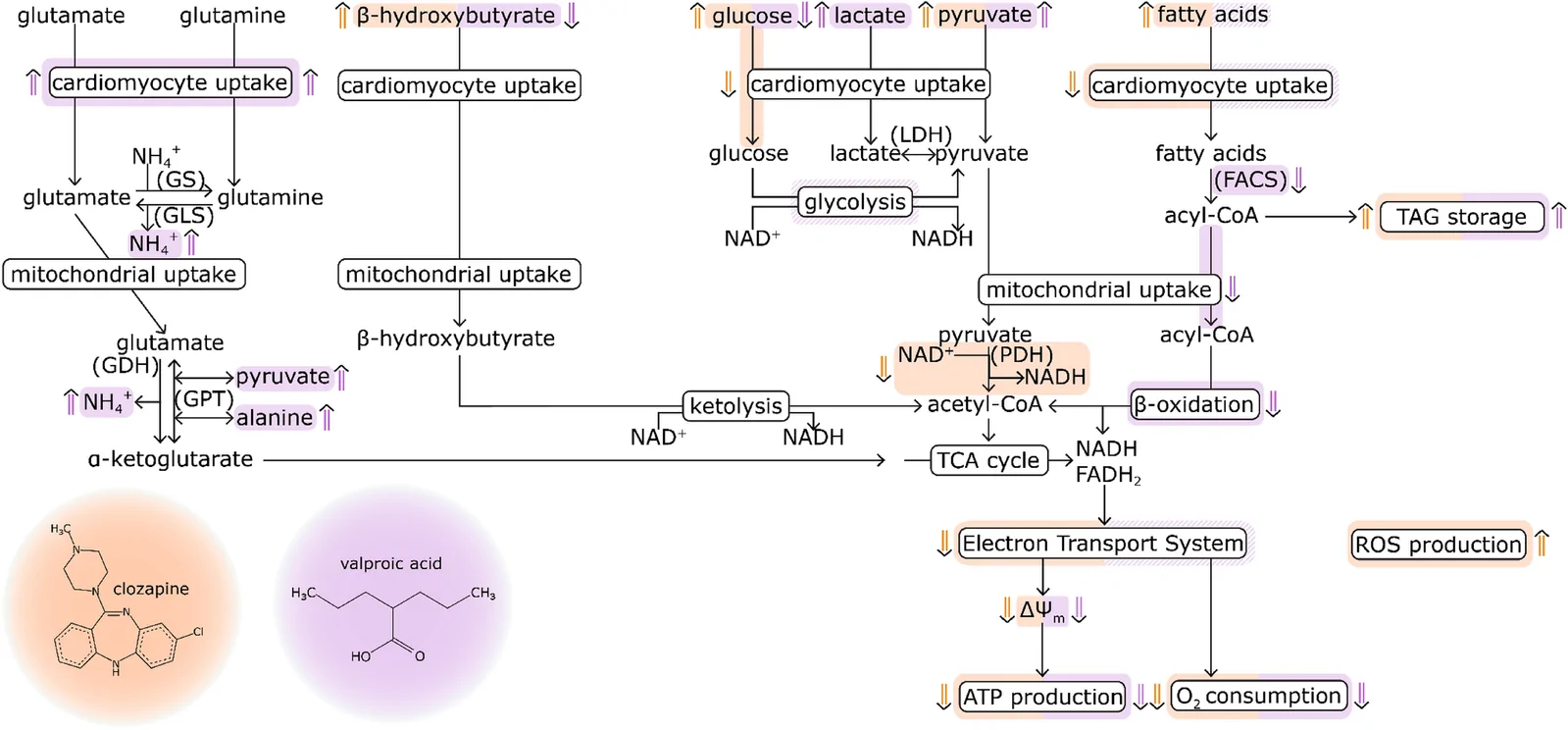

## Introduction

An estimated 24.4% of those first diagnosed with schizophrenia are deemed ‘treatment-resistant’ after failing to respond to or tolerate at least two different antipsychotics at moderate doses for a reasonable period [1, 2]. Compared to treatment-responsive patients, those with treatment-resistant schizophrenia tend to experience more severe symptoms, as well as greater cognitive and psychosocial impairment [3]. Clinical practice guidelines unanimously recommend the prescription of clozapine (CLZ) in treatment-resistant schizophrenia, as it produces a clinically meaningful reduction in symptoms in approximately 40% of these patients [4, 5]. Furthermore, despite being given to those with more severe illness, CLZ has been associated with better outcomes regarding hospitalisation, discontinuation, and symptom management when compared to other antipsychotics [6]. CLZ is also the only antipsychotic that has been shown to decrease the risk of suicidal behaviour in patients [7–9]. In recognition of its clinical importance, CLZ was added to the World Health Organisation’s *Essential Medicines List* in 2013 [10]. Clinical practice guidelines often recommend that CLZ therapy be initiated with some urgency after treatment-resistance has been identified, as deferring treatment is associated with impaired effectiveness [11–13]. Despite this, global delays in the initiation of CLZ range from 1.1 to 9.7 years [14].

The reluctance to initiate CLZ therapy and its designation for use *only* in treatment-resistant schizophrenia can be attributed to its often life-threatening adverse effects (AEs), including cardiotoxicities. The spectrum of cardiotoxicities associated with CLZ is broad, including myocardial infarction, myocarditis, cardiomyopathy and heart failure, as well as arrhythmias and sudden cardiac death [15–17]. Common CLZ-related cardiac AEs include sinus tachycardia, with incidences as high as 68%, as well as orthostatic hypotension and syncope, which are reported in approximately 11% of patients [18, 19]. These AEs are dose-dependent and can be attributed to the known pharmacological actions of CLZ on the autonomic nervous system, including the elevation of plasma catecholamines, and antagonism at α-adrenergic receptors [20–23]. In contrast, other CLZ-associated cardiotoxicities are neither dose-dependent nor predictable, and the cellular mechanisms underlying their occurrence are unknown. The uncertainty surrounding their aetiology translates into a lack of clarity surrounding their clinical prevention, detection, and management [24]. Regardless of how cardiotoxicity presents, the only constant recommendation is that CLZ therapy be withdrawn [25]. Patients may also be provided with supportive care, which can involve the empirical use of heart failure treatments and/or corticosteroids [26–28].

The potential pathophysiological mechanisms underlying the development of CLZ-associated cardiotoxicities have been the focus of several reviews [29, 30]. The most often-proposed mechanisms include a drug-induced hypersensitivity reaction, the induction of cardiac cell death pathways, prolonged increases in cardiac workload as a consequence of elevated plasma catecholamines, and the induction of cardiac oxidative stress. Recently, there has been an emerging appreciation for a potential role of the induction of cardiomyocyte mitochondrial dysfunction [31, 32]. This is largely proposed in the context of oxidative stress caused by the production of reactive oxygen species (ROS) during the cellular metabolism of CLZ. This hypothesis is based on the plasma metabolic profile of CLZ, and the observation that the production of CLZ’s major metabolites, N-desmethylclozapine and clozapine-N-oxide, is significantly shifted in favour of the N-oxide in patients with myocarditis [33]. The N-oxide metabolite itself is not especially reactive, but has been associated with REDOX cycling in vitro, wherein the cyclical univalent REDOX reactions that convert the N-oxide metabolite to its parent compound and back are associated with the reduction of molecular oxygen to superoxide and secondary ROS such as hydrogen peroxide [34, 35]. CLZ can also be bioactivated to reactive iminium and nitrenium ions that can induce oxidative cellular damage alone. These two forms of bioactivation have been observed in murine cardiac microsomes and mitochondria and are thought to cause cardiac mitochondrial damage and dysfunction [36, 37]. Mitochondrial dysfunction has been associated with a diverse array of cardiac pathologies and may present a unifying mechanism behind the broad spectrum of CLZ-associated cardiotoxicities. For instance, mitochondria play a key role in the coordination of cell death pathways that may precede the aberrant inflammatory signalling causing myocarditis [38]. Mitochondrial energy supply is also essential to fuel the cyclical contraction and relaxation of the myocardium through cardiomyocyte excitation–contraction coupling, so that energetic insufficiency can precede the development of cardiomyopathies, dysrhythmias, and heart failure, especially during periods of elevated energetic demand brought on by high plasma catecholamine levels [39].

CLZ also appears to have broader metabolic effects, including a strong association with dyslipaemia and weight gain, as well as an increased risk of type 2 diabetes [40, 41]. These might independently increase the risk of patients developing cardiac AEs such as myocardial infarction. They may also be involved in exacerbating the effects of CLZ on cardiomyocyte energetics by impacting substrate supply and/or storage. There has been some suggestion that diabetic and dyslipidaemic CLZ-taking patients may also be at an increased risk of developing cardiotoxicities [42–45]. Additionally, there appears to be an increased incidence of obesity among fatal compared to non-fatal cases of CLZ-induced myocarditis [46–48]. This might indicate that CLZ’s metabolic and cardiac AEs share important mechanisms and that perturbations in both cellular and systemic energetics are common to different forms of CLZ-associated cardiotoxicity.

Sodium valproate comedication is another important risk factor in the development of cardiotoxicities during CLZ treatment [44]. Sodium valproate, or valproic acid (VPA), is an anti-epileptic and mood stabiliser that is commonly prescribed to augment antipsychotic therapy in schizophrenia, providing significant additional benefit in terms of symptom severity [49, 50]. VPA may also be prescribed as a prophylactic measure to manage CLZ-associated seizures [51, 52]. VPA alone has not been strongly associated with any cardiac AEs, apart from tachycardia and haemodynamic instability in overdose [53]. The role of VPA in exacerbating the risk of CLZ-associated cardiotoxicities is therefore unlikely to be a simple additive effect.

VPA alone is strongly associated with hepatic injury and failure [54–56]. This is thought to be related to oxidative stress during first-pass drug metabolism, wherein a proportion of VPA is bioactivated by cytochrome P450 (CYP450) and mitochondrial β-oxidation enzymes to form the reactive metabolites 4-ene-VPA-CoA and 2,4-diene-VPA-CoA, respectively [57, 58]. The mitochondrial metabolism of VPA also has negative consequences on hepatic adenosine triphosphate (ATP) production, by inhibiting fatty acid (FA) oxidation, additionally leading to the accumulation of cellular FAs and hepatic steatosis [53, 59, 60]. The well-established nature of VPA as a mitochondrial toxicant makes its lack of cardiac AEs especially surprising, considering the heart’s high energy requirements and considerably lower antioxidant capacity [61]. This might indicate that VPA is not bioactivated in cardiomyocytes as it is in hepatocytes, either due to differences in cellular xenobiotic metabolism or in drug uptake, though it has been shown to accumulate in both organs in primates [62]. VPA’s primary role in increasing the risk of CLZ-associated cardiotoxicities may lie in its alteration of CLZ metabolism, as it has been shown to alter CLZ’s plasma metabolic profile in patients [63]. If this also occurs in cells, VPA comedication would augment CLZ-related REDOX cycling and oxidative stress. However, those CYP450 enzymes associated with CLZ REDOX cycling and the bioactivation of VPA are expressed in both the heart and liver, though not to the same extent [64]. If altered xenobiotic metabolism was solely responsible for these adverse effects, CLZ and VPA might both be expected to cause oxidative stress in both organs either alone or in combination. It might also indicate that cardiomyocytes and/or their mitochondria are resistant to these effects, or able to adapt to them. For example, while cardiomyocytes are typically reliant on FA oxidation to produce ATP, they can metabolise other fuel sources in the face of increasing energy demand or changes in substrate supply [65, 66]. If VPA does impair cardiac FA oxidation, it is possible that this would have little to no clinically detectable effect on contractile function but could pre-sensitise the heart to the negative oxidative and energetic effects of CLZ. Additionally, VPA is associated with metabolic AEs, including weight gain, dyslipidaemia, and hyperinsulinaemia [67, 68]. As with CLZ, these actions of VPA may independently increase the risk of cardiac AEs.

Considering the above, this review aimed to summarise the existing evidence for the effects of CLZ and VPA on cellular and systemic energetics, and to propose how these might contribute to the development of CLZ-associated cardiotoxicities. In writing this review, we hypothesise that CLZ and VPA act on separate but converging pathways in the production of ATP by cardiac mitochondria, resulting in synergistic cardiac dysfunction. To that end, the effects of both drugs on mitochondrial function and substrate metabolism were summarised. While evidence from all organ systems is presented, the focus of this review is to consider how these could contribute specifically to cardiac dysfunction. To assist the reader with critically assessing the quality of the evidence presented, key results as well as drug exposure concentrations and periods are summarised in Tables 1, 2, 3, and 4.

## Mitochondrial Dysfunction

### Mitochondrial Membrane Potential and ATP Production

The hydrolysis of ATP is a highly favourable reaction, utilised by cardiomyocytes to fuel the endergonic processes that facilitate their cyclical contraction and relaxation. Most of the ATP within the heart is produced through oxidative phosphorylation (OXPHOS) within mitochondria [69]. OXPHOS refers specifically to the combined work of the electron transport system (ETS) to produce an electrochemical gradient across the inner mitochondrial membrane (the mitochondrial membrane potential; ΔѰ_m_), and the utilisation of this gradient by the F_1_F_0_ ATPase to produce ATP. Both CLZ and VPA have been shown to impair OXPHOS through the depolarisation of the ΔѰ_m_ and the impairment of ATP production. Investigations in isolated rat cardiomyocytes provide the most direct evidence for ΔѰ_m_ depolarisation following CLZ exposure [70–72], though this has also been observed in a number of cell lines including neural, adipocyte, myoblast, hepatocyte, and monocyte cell lines [73] as well as in a differentiating liposarcoma line and human blood–brain barrier-derived endothelial cells [70–72, 74, 75]. Where investigated, CLZ exposure has been associated with decreased ATP levels and an increased ADP/ATP ratio [73, 75, 76]. However, it must be borne in mind that all studies considered only the acute effects of CLZ and therefore employed exposure concentrations well above the therapeutic plasma concentration range [77]. Whilst these effects could be speculated to accumulate over time at lower concentrations, their time- and concentration-dependence has not been determined.

While there has not been any evidence for its effect in cardiac experimental models, VPA has been shown to cause ΔѰ_m_ depolarisation in neural, glial, and cervical cell lines, as well as in rat kidney and brain mitochondria [78–84]. VPA exposure has also been associated with ATP depletion in mouse brain and rat kidney, as well as neural and hepatic cell lines, though this was not demonstrated following a shorter period of exposure in isolated hepatocytes [82, 85–88]. The exposure concentrations used in these studies do encompass the therapeutic plasma concentration range [77], though much like with CLZ these changes were mostly observed following acute drug exposure. The lack of direct evidence limits any conclusions that can be drawn, although the range of cell lines and organ systems involved is diverse enough to expect the same might occur in cardiac mitochondria should they be exposed to VPA. Considering specifically those studies where hepatic cell lines were used, VPA and CLZ exposure appeared to be independently detrimental to the maintenance of ΔѰ_m_ and ATP production, suggesting that there may be some convergent effect of both drugs in causing mitochondrial dysfunction [73, 88]. It is unclear if this would be the case in cardiomyocytes as well, since the mechanisms behind these changes in ΔΨ_m_ and ATP production have not been determined.

### The Electron Transport System

The ETS can be conceptualised as a network containing several electron transferring pathways. The electron carrier nicotinamide adenine dinucleotide (NADH) initiates respiratory complex I (CI)-linked electron transfer, whereas flavin adenine dinucleotide (FADH_2_) is oxidised within the prosthetic groups of CII, the electron-transferring flavoprotein complex, and the glycerol-3-phosphate dehydrogenase [89]. These pathways converge at the mobile carrier ubiquinone and terminate with the two-electron reduction of O_2_ at CIV [90]. Changes in mitochondrial respiration and the activity of ETS enzymes would be the most direct means of altering ΔΨ_m_ and ATP production.

Indirect evidence from a neuronal cell line and isolated rat brain mitochondria suggests that CLZ decreases CI activity [76, 91]. Similarly, in an endothelial cell line, CLZ was reported to impair both CI and CIII activity [75, 92]. In differentiating adipocytes, changes in respiration following CLZ exposure were not observed unless normalised to the number of mitochondria, in which case respiration was increased, seemingly due to increased expression of ETS complexes that may have compensated for mitochondrial damage and inefficient OXPHOS [74]. More direct evidence from isolated rat ventricular cardiomyocytes suggests that CLZ inhibits CII enzyme activity [72], and in human cardiac mitochondria, we have shown that acute CLZ exposure impairs both CI- and CII-mediated respiration [93]. It therefore seems likely that CLZ impairs OXPHOS, at least to some extent, via inhibitory actions on the ETS. Importantly, as with evidence from CLZ’s effect on ΔΨ_m_ and ATP production, these studies were conducted following acute exposure to supra-therapeutic concentrations of CLZ. However, in peripheral blood mononuclear cells from CLZ-treated patients, the activities of individual complexes and the rate of CI-, CII-, and glycerol-3-phosphate dehydrogenase-linked respiration were not significantly different from those of treatment-naïve patients, indicating that these effects are not relevant in these cells, or at typical plasma concentrations of CLZ [92].

VPA has been reported to impair respiration and decrease the expression of respiratory complexes in a breast cancer cell line, although it increased cellular respiration in mesenchymal stem cells following a lower concentration but longer period of exposure [94, 95]. On the other hand, in human HAP1 cells, VPA seemed to impair respiration, while increasing respiratory complex activity at supratherapeutic concentrations of exposure [96]. VPA also appears to impair respiration in yeast and zebrafish embryos at lower and even subtherapeutic concentrations of exposure [97, 98]. In isolated pig brain mitochondria, VPA had no effect on respiration, though CI and CIV activity were impaired at higher concentrations [99, 100]. Conversely, VPA appeared to increase CII activity in rat brain [101]. Similarly, VPA increased maximal respiration and the transcription of respiratory complexes in neuronal and microglial cell lines [102]. However, it appeared to slightly decrease cellular respiration in isolated retina as well as CI- and CII-linked respiration in cerebral mitochondria [103]. VPA’s effects on hepatocytes are much clearer, with impaired respiration and mitochondrial uncoupling consistently reported in hepatic cell lines as well as isolated rat liver mitochondria [88, 104–108]. As with studies of ΔΨ_m_ and ATP production, there is little direct evidence from cardiac models regarding VPA’s effect on the ETS. In human cardiac mitochondria, VPA may impair respiration and respiratory coupling, though this is not entirely clear, as it may also act as a FA substrate and induce respiration [93]. Further study is required to isolate the specific actions of VPA on mitochondrial respiration in cardiomyocytes and determine how these might contribute to changes in OXPHOS.

### Mitochondrial ROS Production and the Permeability Transition Pore

The ETS is also a major source of ROS under physiological conditions, through ‘electron leak’ and the resultant incomplete reduction of O_2_ to produce superoxide [109]. Mitochondria can also become a source of cellular ROS through the opening of the mitochondrial permeability transition pore (mPTP). The molecular identity of the mPTP, as well as its role in normal physiology, are contentious [110]. However, most agree that mPTP opening is mediated by changes in mitochondrial Ca^2+^ levels, and that its sensitivity to Ca^2+^ can be increased when mitochondrial ADP is depleted, the ΔѰ_m_ is depolarised, or ROS production is increased. Opening of the mPTP can further induce ROS production due to the resultant depolarisation of the ΔѰ_m_ and the oxidation of the NADH and NADPH pools that contribute to antioxidant defences [111].

CLZ is often associated with increased mitochondrial ROS production. In differentiating mouse and human liposarcoma cell lines, CLZ increased mitochondrial ROS production, but appeared to decrease cytosolic ROS production [74, 112]. This has not been reported in any other model, for example, in isolated submitochondrial particles from rat hippocampi, CLZ induced superoxide production but also increased markers of protein and lipid oxidative damage in brain tissue [113]. More directly, a group from Ardabil University of Medical Sciences has repeatedly shown in isolated rat cardiomyocytes that CLZ exposure promotes cellular ROS production, associated with increased lipid peroxidation and decreased glutathione content [70, 71]. Cardiomyocyte oxidative stress was accompanied by ΔѰ_m_ collapse in these studies. They later showed that, in isolated rat heart mitochondria, CLZ exposure caused a concentration-dependent increase in mitochondrial swelling, which might be indicative of ROS-related mPTP opening [72]. However, another group has shown that the same concentrations of CLZ did not induce mPTP opening over a shorter period of exposure, though the formation of its N-oxide metabolite relative to its N-desmethyl metabolite was already increased in cardiac mitochondria [36]. As previously described, the N-oxide metabolite and CLZ itself have been associated with a concentration-dependent increase in the CYP450-catalysed production of ROS in a cell-free system [35]. Increased mitochondrial ROS production following CLZ exposure has also been observed in human stem cell-derived cardiomyocytes, though whether this was related to mPTP opening or bioactivation was not investigated [114]. Regardless, the observation that there is cardiac microsomal and mitochondrial bioactivation certainly seems a compelling primary effect of this drug, which may precede the impairment of OXPHOS and/or opening of the mPTP [36]. While the authors found that this occurs in hepatic microsomes and mitochondria as well, the heart is likely to be more vulnerable to increased REDOX cycling owing to a lower antioxidant capacity and lower reserve due to its relatively higher mitochondrial density, cellular O_2_ concentration and therefore ROS production under physiological conditions [61].

The effect of VPA on mitochondrial ROS production appears to be tissue specific. In rat brain submitochondrial particles, VPA had no effect on superoxide production [115]. This aligns with an apparent lack of effect on respiration in brain mitochondria, described previously. In mitochondria isolated from the kidney of VPA-exposed rats, ROS production was elevated, aligning with the observed ΔѰ_m_ depolarisation and increased sensitivity to elevated mitochondrial Ca^2+^ concentrations that might induce mPTP opening [82]. In hepatic cell lines and isolated rat liver mitochondria, the general consensus appears to be that VPA increases mitochondrial ROS production and impairs antioxidant capacity [88, 105, 116–118]. However, in isolated mouse liver mitochondria, VPA instead decreased mitochondrial ROS production [119]. In a human hepatic cancer (Huh7) cell line, a similar extent and duration of VPA exposure also decreased mitochondrial ROS production, although cytosolic ROS production was still increased [120]. This might be expected, especially since VPA appears to impair respiration and respiratory coupling in hepatocytes, which decreases mitochondrial ROS production by decreasing the ΔѰ_m_ and the rate of ‘electron leak’ as it relates to electron transfer [121]. However, as previously described, VPA can also be bioactivated by CYP450 and mitochondrial β-oxidation enzymes [57, 58]. In vitro work in both hepatic and non-hepatic cell lines, and in zebrafish embryos, has consistently demonstrated that VPA exposure increases cellular ROS production [84, 96, 98, 122]. VPA may therefore have contradictory effects on ROS levels, depending on which of these pathways are dominant, so that its effects are tissue- and species-specific. In cardiomyocyte cell lines, VPA exposure did not have any effect on mitochondrial ROS production, so it is possible that in the heart VPA is not bioactivated as it is in the liver [123]. As alluded to previously, this might be due to tissue-specific differences in the expression of CYP450 enzymes, which are less abundant in the heart [64]. However, it is important to note that direct evidence for VPA’s effect in cardiomyocytes and their mitochondria is limited, and the only evidence presented here involved cell lines exposed to subtherapeutic concentrations of the drug over a short period (Table 1).

**Table 1:** Summary of evidence for clozapine (CLZ)- and valproate (VPA)-related mitochondrial dysfunction

First author [References]	Experimental model	Exposure	Relevant results
**ΔѰ**_**m**_ ** and ATP production**			
*Clozapine*			
Ahangari [70]	Isolated cardiomyocytes (male Wistar rats)	50 µM, 4 h	ΔѰ_m_ depolarisation
Bakhshii [71]	Isolated cardiomyocytes (male Wistar rats)	50 µM, 4 h	ΔѰ_m_ depolarisation
Hafez [72]	Isolated cardiomyocytes (male Wistar rats)	50 µM, 6 h	ΔѰ_m_ depolarisation
Contreras-Shannon [73]	SKNSH cells (human neuroblastoma line)	10/20/50 µM, 24 h	ΔѰ_m_ depolarisation (≥ 20 µM)
	3T3-L1 cells (mouse preadipocyte line)	25/50/75 µM, 24 h	ΔѰ_m_ depolarisation (75 µM), ATP depletion (≥ 50 µM)
	C2C12 cells (mouse skeletal muscle myoblast line)	25/50/75 µM, 24 h	ΔѰ_m_ depolarisation (≥ 50 µM)
	FL83B cells (mouse embryonic hepatocyte line)	25/50/75 µM, 24 h	ΔѰ_m_ depolarisation (75 µM), ATP depletion (≥ 50 µM)
	RAW 264.7 cells (mouse monocyte line)	25/50/75 µM, 24 h	ΔѰ_m_ depolarisation and ATP depletion (≥ 25 µM)
Elmorsy [75]	Human blood brain barrier-derived endothelial cells	~4–600 µM, 24 h	ATP depletion (IC_50_ ~ 30 µM), ΔѰ_m_ depolarisation (30 µM)
Fiorani [74]	SW872 cells (human liposarcoma line)	5/10/15 µM, 72 h	ΔѰ_m_ hyperpolarisation (≥ 5 µM), ATP depletion (15 µM)
Ota [76]	PC12 cells (rat medullary pheochromocytoma line)	50 µM, 24 h	Unchanged ΔѰ_m_, increased ADP/ATP ratio with normal glucose. With high glucose ADP/ATP restored and hyperpolarised ΔѰ_m_
*Valproate*			
Tikhonova [83]	Male Wistar rats	100/250/500/1000 mg/kg, Acute	Decreased erythrocyte ATP levels and increased ADP and AMP levels (≥ 500 mg/kg)
Coudé [85]	Isolated hepatocytes (male Wistar rats)	0.1–5 mM, 60 min	Unchanged cellular ATP content
Komulainen [88]	HepG2 cells (human hepatocellular carcinoma line)	0.5/1/2 mM, 24/48/72 h	ΔѰ_m_ depolarisation (≥ 1 mM) at 24–48 h, ATP depletion (2 mM @ 24 h, ≥0.5 mM @ 48–72 h)
Heidari [82]	Isolated kidney mitochondria (male Sprague–Dawley rats)	250/500 mg/kg/day (i.p.), 15 days	ΔѰ_m_ depolarisation, ATP depletion
Johannessen [87]	Female Hsd-Ola ICRF mouse brain extracts	400 mg/kg (i.p.), 25/45 min	ATP depletion @ 25 min, recovered by 45 min
Chaudhary [78]	Brain synaptosomes (male Wistar rats)	5 mM, 2 h	ΔѰ_m_ depolarisation
Chen [79]	Isolated microglial cultures (Fisher F344 rat pups)	0.3/0.6/1.2 mM, 24 h	ΔѰ_m_ depolarisation
Fu [80]	U87MG, SF295, T98G cells (human glioblastoma lines)	0.25/0.5/1.0/1.5 mM, 96 h	ΔѰ_m_ depolarisation
Jang [86]	SH-SY5Y cells (human neuroblastoma line)	1/5/10 mM, 4 h	ΔѰ_m_ depolarisation and ATP depletion (≥ 5 mM)
Horonyova [96]	HAP1 cells (near-haploid chronic myeloid leukemia-derived line)	0/0.06/0.6 mM, 24 h	ΔѰ_m_ hyperpolarisation and increased ATP production (0.06 mM)
Han [81]	HeLa cells (human cervical adenocarcinoma line)	1/3/5/10/15 mM, 24 h	ΔѰ_m_ depolarisation (≥ 3 mM)
Zhou [84]	L02 cells (HeLa derivative line)	6 mM, 24 h	ΔѰ_m_ depolarisation
Du [94]	MCF-7 and MDA-MB-231 cells (human mammary gland adenocarcinoma lines)	0, 2, 4 mM 24 h	ΔѰ_m_ depolarisation
Salsaa [97]	BY4741 MATa yeast	0.6 mM, 1 h	ΔѰ_m_ depolarisation
**Electron transport system**			
*Clozapine*			
Groenewald [93]	Permeabilised fibres (human right atrial appendage)	15 µM, 30 min	Decreased CI- and CII- mediated state 3 O_2_ consumption
Hafez [72]	Isolated cardiac mitochondria (male Wistar rats)	5–100 µM, 1 h	Decreased CII activity (≥ 20 µM)
Casademont [92]	Peripheral blood mononuclear cells from schizophrenic patients	Variable doses, Mean period 44.5 months	Unchanged CI, CII, CIII, or CIV activity
Elmorsy [75]	Human blood brain barrier-derived endothelial cells	~30 µM, 24 h	Decreased CI and CIII activity
Burkhardt [91]	Rat brain mitochondria	0–300 µM, 3 min	Decreased CI activity (≥ 90 µM). Decreased CIII but not CIV activity (300 µM)
Ota [76]	PC12 cells (rat medullary pheochromocytoma line)	50 µM, 24 h	Decreased CI activity
Fiorani [74]	SW872 cells (human liposarcoma line)	15 µM, 72 h	Increased CI, CII, CIV, and CIV protein expression. Unchanged O_2_ consumption/cell, increased O_2_ consumption/mitochondria
*Valproate*			
Groenewald [93]	Permeabilised fibres (human right atrial appendage)	6 mM, 30 min	Decreased CII-mediated state 3 O_2_ consumption and CI OXPHOS efficiency
Pirozzi [105]	HepG2 cells (human hepatocellular carcinoma line)	2 mM, 48 h	Decreased basal, state 3, and uncoupled respiration
	Rat hepatocytes (male Wistar rats)	1 mM, 24 h	Decreased CII-linked state 3 and 4 respiration
Komulainen [88]	HepG2 cells (human hepatocellular carcinoma line)	0.5/1/2 mM, 24/48/72 h	Decreased respiration at 24 h (≥ 1 mM) and 48 h (2 mM), recovered by 72 h
Ponchaut [106]	Hepatic mitochondrial fraction (male Wistar rats)	1% (w/w), 75 days	Increased CII expression. Decreased CI- and CII-linked state 3 respiration. Decreased CIV activity
Jimenez-Rodriguezvila [104]	Isolated hepatic mitochondria (female Wistar rats)	60/120 mg/kg/day, 4/18 weeks	Decreased P:O ratio (18 weeks only)
Natarajan [117]	Isolated hepatic mitochondria (female Wistar rats)	700 mg/kg/day, 5 days	Decreased respiratory control ratio
Jafarian [116]	Isolated hepatic mitochondria (male Sprague–Dawley rats)	25/50/100/200 µM, 5–60 min	Decreased CII activity (≥ 100 µM)
Veitch [108]	Isolated hepatic mitochondria (male Wistar rats)	1 mM/ 0.001–1 mM concentration–response	Decreased state 3 respiration. State 3 respiration with pyruvate/malate more sensitive to inhibition than with palmitoylcarnitine or glutamate
Chalifoux [119]	Isolated hepatic mitochondria (male C57BL6N mice)	10 mM, acute	Decreased respiratory control ratio
Rumbach [107]	Isolated hepatic and cerebral mitochondria (male Wistar rats)	200 mg/kg, acute 600 mg/kg/day, 5 days	Decreased CI- and CII-mediated respiration – less severe in cerebral mitochondria
Zager [103]	Isolated retina (New Zealand White rabbits)	5 mM, Acute	Slight decrease in cellular respiration
Feier [101]	Brain homogenate (male Wistar rats)	400 mg/kg/day (i.p.), 7 days	Increased CII activity
Hroudova [100]	Isolated pig brain mitochondria	5 mM, 30 min	Decreased CI and CIV activity
Cikánková [99]	Isolated pig brain mitochondria	50 µM, 30 min	Unchanged CI, CII+III, CIV activity
Bortolasci [102]	NT2-N cells (human embryonal carcinoma-derived neuronal line) C8-B4 cells (mouse microglial line)	0.5 mM, 24 h	Increased CI and CIV gene expression and uncoupled respiration in NT2-N cells. Increased CI-V expression and maximal respiratory capacity in C8B4 cells
Camussi [98]	AB zebrafish embryos	25/50 µM, 114 h	Decreased basal and maximal respiration
Killer [95]	Human mesenchymal stem cells	1 mM, 6 days	Increased rate of cellular respiration
Horonyova [96]	HAP1 cells (near-haploid chronic myeloid leukemia-derived line)	0/0.06/0.6 mM, 24 h	Increased state 3 O_2_ consumption, increased CI expression, and increased CI-III activity (≥ 0.06 mM). Decreased uncoupled- and increased state 4 O_2_ consumption, decreased CI expression but unchanged activity (0.6 mM)
Du [94]	MCF-7 and MDA-MB-231 cells (human mammary gland adenocarcinoma lines)	0/2/4 mM, 24 h	Decreased basal (≥ 2 mM) and uncoupled (2 mM) O_2_ consumption in MCF-7 cells. Biphasic concentration–response in MDA-MB-231 cells
Salsaa [97]	BY4741 MATa yeast	1 mM, 2–10 min	Decreased basal respiration, unchanged CIII and CIV activity
**Mitochondrial ROS production/mPTP opening**			
*Clozapine*			
Vaziri [114]	iPSC-derived human cardiomyocytes	2.8 µM, 24 h	Increased mitochondrial ROS from myocarditis and control patient cells, not in cells from CLZ-tolerant patients
Arzuk [36]	Isolated cardiac and hepatic mitochondria and microsomes (male Swiss albino mice/Wistar albino rats)	100 µM, 1 h	Lower concentrations of CLZ metabolites in heart vs liver mitochondria and microsomes, except GSH conjugate of reactive metabolite. Similar amount of N-desmethyl and CLZ N-oxide in and outside mitochondria, lower amount of CLZ and GSH-conjugate in mitochondria
Hafez [72]	Isolated cardiac mitochondria (male Wistar rats)	5–100 µM, 1 h	Mitochondrial swelling (concentration-dependent)
Polydoro [113]	Brain submitochondrial particles (male Wistar rats)	25 mg/kg/day, 28 days	Increased superoxide production
Fiorani [74]	SW872 cells (human liposarcoma line)	5/10/15 µM, 72 h	Increased mitochondrial superoxide production
Blandino [112]	SW872 cells (human liposarcoma line)	15 µM, 72 h	Increased mitochondrial superoxide production
*Valproate*			
Ahn [123]	AC16 cells, (human cardiomyocyte-fibroblast hybrid line) H9c2 cells (embryonic rat cardiac line)	1/10/100 µM, 24 h	Unchanged mitochondrial superoxide production
Tang [118]	Male Sprague–Dawley rats	100 mg/kg/day (i.p.), 5 days	Unchanged hepatic total GSH, depletion of mitochondrial GSH
		200 mg/kg/day (i.p.), Acute	Depletion of total hepatic and mitochondrial GSH
Pirozzi [105]	HepG2 cells (human hepatocellular carcinoma line)	2 mM, 48 h	Decreased SOD2 expression and activity, decreased aconitase activity
	Isolated hepatocytes (male Wistar rats)	1 mM, 24 h	Decreased SOD activity
Gai [120]	Huh7 cells (human hepatic carcinoma line)	2 mM, 24 h	Increased total and cytosolic ROS production. Decreased mitochondrial ROS production
Komulainen [88]	HepG2 cells (human hepatocellular carcinoma line)	0.5/1/2 mM, 24/48/72 h	Elevated mitochondrial ROS (2 mM, 72 h only). Decreased SOD2 expression
Chalifoux [119]	Isolated hepatic mitochondria (male C57BL6N mice)	0.1/1/5/10/25 mM, acute	Decreased mitochondrial ROS production (≥ 5 mM)
Jafarian [116]	Isolated hepatic mitochondria (male Sprague–Dawley rats)	25/50/100/200 µM, 1 h	Increased mitochondrial ROS production, lipid peroxidation (≥ 50 µM), and GSH depletion (200 µM)
Natarajan [117]	Isolated hepatic mitochondria (female Wistar rats)	700 mg/kg/day, 5 days	Increased mitochondrial swelling and decreased Ca^2+^ influx
Heidari [82]	Isolated kidney mitochondria (male Sprague–Dawley rats)	250/500 mg/kg/day, 15 days	Increased mitochondrial reactive species production, lipid peroxidation, and GSH depletion
Valvassori [115]	Brain submitochondrial particles (male Wistar rats)	400 mg/kg/day, 13 days	Unchanged superoxide production
Horonyova [96]	HAP1 cells (near-haploid chronic myeloid leukemia-derived line)	0/0.06/0.6 mM, 24 h	Elevated cellular ROS and superoxide (0.6 mM), increased mitochondrial Ca^2+^ (0.06 mM)

## Deranged Substrate Metabolism

The tricarboxylic acid (TCA) cycle serves as an important source of the reducing equivalents required for CI- and CII-linked electron transfer. The cycle is initiated by the condensation of oxaloacetate and acetyl-CoA, with later reactions producing CO_2_, GTP, and the reducing equivalents NADH and FADH_2_, before regenerating oxaloacetate. TCA cycle intermediates can serve as biosynthetic precursors in cataplerotic reactions, but must be replenished by anaplerotic reactions to facilitate continued OXPHOS. This would also require the continued supply of acetyl-CoA through the oxidation of fuel substrates including FAs, carbohydrates, ketones, and amino acids.

### Fatty Acid Metabolism

FA oxidation accounts for the majority of ATP production in cardiomyocytes [124, 125]. These are supplied to the heart bound to albumin or esterified as triacylglycerol (TAG) in circulating lipoproteins [126, 127]. The uptake of FAs by the heart is thought to be mediated by the sarcolemmal isoform of the FA binding protein (FABP), which concentrates FAs prior to their diffusion or transport across the sarcolemma by the FA translocase (FAT/CD36) or FA transport protein 1/6 [128]. Cardiac TAG uptake comes primarily from chylomicrons being hydrolysed by lipoprotein lipase on the surface of endothelial cells to release FAs [127].

There have been no mechanistic studies to investigate whether cardiac FA uptake is altered by CLZ beyond a single investigation in which it was shown to increase FAT/CD36 expression in a macrophage cell line, possibly contributing to the formation of foam cells that exacerbate atherosclerosis progression even at subtherapeutic concentrations [129]. In schizophrenic patients treated with CLZ, the diagnosis of metabolic syndrome was correlated with changes in the serum levels of FABP4, which is thought to be released by adipocytes during lipolysis [130]. However, in cultured human-derived adipocytes, CLZ exposure was not associated with changes in triglyceride content or FABP4 expression at therapeutic concentrations [131]. CLZ is, however, strongly associated with elevations in total plasma cholesterol and TAG in schizophrenic patients [132–139]. In rats, CLZ also significantly increased serum FA concentrations, within 15 min of administration [140, 141]. This would likely lead to an increase in cardiac FA uptake, which approximately correlates with plasma concentrations, though there is no direct evidence for this being the case [142].

VPA treatment is less often associated with hyperlipidaemia than CLZ [143], with some in fact reporting a decrease in plasma cholesterol [144, 145], and others reporting no change in serum cholesterol, TAG, or FA levels in epileptic patients as well as rodent models [146–149]. However, VPA has been shown to increase the surface expression of FAT/CD36 and FABP4 in hepatocytes, which was associated with lipid accumulation and increased lipid droplet formation, although a higher concentration and duration of exposure decreased FABP expression in other hepatic cell lines while still increasing CD36 expression [150–153]. The development of obesity in VPA-treated epileptic patients has also been associated with single-nucleotide polymorphisms in the gene encoding FAT/CD36 [154]. In plasma from diabetic patients, free VPA levels were higher than in non-diabetic controls, which was associated with increased serum FA levels [155]. This could be indicative of competition between VPA and endogenous FAs for binding sites on albumin, which appear to have a lower affinity for VPA. This might be protective in one sense, as VPA could buffer the levels of free FAs in the serum by relinquishing its binding sites on albumin. On the other hand, an increase in serum FA levels would therefore increase VPA levels as well. However, since there is no consensus on the effect of VPA on FA supply, and the only evidence for its modifying cellular FA uptake comes from hepatic cell lines, it is difficult to make any prediction regarding its possible effect in cardiomyocytes.

Within the cytosol FAs are converted to CoA esters by fatty acyl-CoA synthetases (FACS), which can then be shunted to catabolic or anabolic pathways, while still maintaining a driving force for FA uptake [156, 157]. However, there is an apparent threshold plasma FA concentration below which uptake is minimal [158, 159], and the little uptake that does occur appears to be largely esterified to form TAG [160]. Otherwise, approximately 10–30% of cardiac FA uptake is esterified, while the remainder is immediately oxidised [161, 162]. Prior to their oxidation, acyl-CoA molecules must be imported into the mitochondrial matrix via the carnitine shuttle [163]. This involves the outer membrane carnitine palmitoyltransferase (CPT) I, the carnitine-acylcarnitine translocase of the inner membrane, and CPT II on the matrix side of the inner membrane [164, 165]. Carnitine levels in the cytosol and matrix are both important in facilitating this process. Mitochondrial FA uptake is further regulated by the allosteric inhibition of CPT I by malonyl CoA, synthesised from acetyl CoA by acetyl-CoA carboxylase (ACC) and degraded by malonyl-CoA decarboxylase [166, 167].

CLZ exposure has been shown to inhibit carnitine uptake in renal and hepatic cell lines, and apparently decreased plasma and hepatic carnitine levels in mice, likely impairing mitochondrial FA uptake [149]. In contrast, CLZ has also been associated with ACC inactivation in rat prefrontal cortex and a mouse skeletal muscle cell line, which could alleviate the inhibition of mitochondrial uptake by malonyl-CoA [168, 169]. However, there has been no direct evidence to suggest that CLZ affects mitochondrial FA uptake in any experimental model. VPA, on the other hand, has been shown to inhibit medium-chain FACS in hepatocytes, thereby impairing their activation and mitochondrial uptake [170]. The conversion of VPA to a CoA ester by FACS also sequesters hepatic CoA, further impairing the activation and mitochondrial uptake of FAs of any length [60, 171, 172]. VPA treatment has also been associated with decreased plasma carnitine concentrations in patients and decreased hepatic carnitine concentrations in mice [60, 146, 172–175]. Furthermore, VPA appeared to decrease CPT I expression and enzyme activity, which was associated with cytosolic lipid accumulation in a hepatic cell line [105, 153, 176]. To date, the effects of CLZ and VPA on cardiac mitochondrial FA uptake have yet to be investigated.

In the mitochondria, fatty acyl-CoA molecules enter the membrane- or matrix- β-oxidation pathways [177–181]. Each cycle of β-oxidation shortens the FA moiety by two carbons and produces one molecule each of acetyl-CoA, FADH_2_, and NADH. Each enzyme involved in β-oxidation is regulated by feedback inhibition from these products, decreasing the rate of FA oxidation during periods of low metabolic demand [182]. There has been very little evidence to suggest that CLZ affects mitochondrial FA oxidation. In mice given a single dose of CLZ during the dark cycle, when the ratio of CO_2_ produced to O_2_ consumed is typically elevated due to normal feeding, theirs instead rapidly dropped [183]. This might be attributed to a systemic shift toward FA oxidation, as the complete oxidation of FAs consumes more O_2_ than carbohydrates. However, this might also be a result of increased serum FAs, which become elevated soon after CLZ administration in rats [140] and would likely upregulate systemic FA uptake and oxidation. In contrast, VPA has been well-established to inhibit β-oxidation enzymes, resulting in increased lipid droplet formation and hepatic steatosis, though this has predominantly been investigated in rodent models [85, 153, 170, 184–187]. The inhibition of β-oxidation enzymes by VPA is relatively weak, and the majority of the inhibitory effect on β-oxidation is attributed to its bioactivation to 4-ene-VPA-CoA and 2,4-diene-VPA-CoA within mitochondria as described previously, which both have more potent inhibitory effects [118, 185, 188]. In the absence of VPA bioactivation, drug exposure might be expected to have a modest inhibitory effect on cardiac mitochondrial FA oxidation. Alone, this may not be functionally significant, but it would likely impair cardiac metabolic flexibility. This has been demonstrated in isolated working rat hearts, where VPA exposure caused a decrease in the rate of FA oxidation in the presence of glucose, and a modest increase in FA oxidation when glucose was replaced with either pyruvate or lactate [189]. VPA was shown to impair cellular pyruvate and lactate uptake in this investigation, facilitating a greater reliance on glucose oxidation. The slight increase in FA oxidation in the absence of glucose was proposed to relate to upregulation via the Randle cycle (described below) due to impaired lactate and pyruvate uptake. VPA did not affect mechanical performance in these hearts unless glucose was replaced with lactate or pyruvate, suggesting that a compensatory increase in glucose oxidation following the inhibition of FA oxidation was sufficiently able to support mechanical performance.

Cellular FA synthesis utilises acetyl-CoA derived from mitochondrial citrate, which is transported into the cytoplasm by the mitochondrial citrate carrier [190]. Here, the ATP citrate lyase catalyses the conversion of citrate and CoA into acetyl-CoA and oxaloacetate. Malate dehydrogenase reduces oxaloacetate to malate, which can be transported back into the mitochondrial matrix as part of a non-canonical TCA cycle that regenerates cytosolic NAD^+^ [191, 192] or be converted to pyruvate by malic enzyme in the cytosol, generating NADPH [193]. The acetyl-CoA produced by ATP citrate lyase is converted to malonyl-CoA in an irreversible and ATP-dependent reaction catalysed by ACC [194–196]. The FA synthase enzyme complex generates palmitate by cyclical condensation, reduction, dehydration, and reduction reactions [197]. FA synthesis can also occur within mitochondria, where each step is catalysed by discrete enzymes rather than a multifunctional enzyme complex, which allows for the production of branched-, saturated- and unsaturated FAs of different lengths [198–200].

CLZ has been associated with ACC inactivation in rat prefrontal cortex and a mouse skeletal muscle cell line [168, 169]. This would be expected to impair malonyl-CoA formation and thus cytosolic FA synthesis while alleviating the inhibition of mitochondrial FA uptake by malonyl-CoA to induce FA oxidation. However, CLZ exposure has also been associated with increased ACC expression in rat liver and adipose stem cells, while in hepatic and glioma cell lines it increased FA synthase transcription and expression, which was associated with the upregulation of FA synthesis and the accumulation of intracellular lipids [141, 201, 202]. The upregulation of hepatic FA synthesis aligns with the observed increase in serum FAs following CLZ administration [140] and would likely have important implications in cardiac FA supply. There is also an emerging appreciation for the roles of both cytosolic and mitochondrial FA synthesis in cardiomyocytes, and their impact on cardiac function [192, 199]. However, changes in cardiac FA synthesis following CLZ exposure have not been investigated to date. ACC inactivation has also been observed in primary human and mouse hepatocytes following VPA exposure [176, 203]. Similarly, VPA exposure was associated with decreased FA synthase transcription, accompanied by suppressed TAG accumulation in adipocyte cell lines [204], although the opposite was observed in isolated rat hepatocytes and a hepatic cell line [143, 153, 176]. In these cells, VPA was shown to impair FA synthesis and decrease citrate content in a concentration-dependent manner [171]. VPA exposure in rats significantly decreased serum levels of oleic acid and cholesterol, further supporting that hepatic FA and lipid synthesis may be impaired [145]. This may explain why VPA is not strongly associated with hyperlipidaemia, causing hepatic steatosis by impairing FA oxidation rather than enhancing FA uptake or synthesis.

Cardiomyocytes are also able to synthesise TAG, which is incorporated in lipid droplet vesicles in the sarcoplasm. This requires the activation of long-chain FAs of either endogenous or exogenous origin by FACS [205]. TAG synthesis involves enzymes located on the outer mitochondrial- and sarcoplasmic reticulum- membranes [206]. FAs derived from endogenous TAG have been shown to be responsible for 36% of the energy expenditure in isolated hearts perfused with glucose-containing buffer solutions, decreasing to 11% with the addition of palmitate [207]. The lipolysis of myocardial TAG also serves as a primary source of long-chain FAs to be oxidised [208]. The synthesis of myocardial TAG is usually increased under conditions of elevated FA supply, such as prolonged fasting, and decreased with increased metabolic demand, as with intense exercise [209–211]. Adrenergic stimulation of cardiomyocytes typically enhances TAG synthesis while simultaneously increasing the rate of its breakdown, enhancing myocardial TAG turnover [212, 213]. Increased myocardial TAG levels have been associated with markers of contractile dysfunction, independent of concurrent comorbidities [209, 214].

CLZ exposure has often been associated with elevated TAG and cholesterol levels, as well as lipid droplet accumulation and expansion in hepatocytes [140, 141, 149, 202, 215], adipocytes [216], and a glioblastoma line [201]. CLZ has also been shown to increase lipogenesis and slow isoproterenol-induced lipolysis in adipocytes [217]. These effects on adipose and hepatic cells could, at least in part, explain CLZ’s association with obesity and hyperlipidaemia in patients, though whether this also occurs in cardiomyocytes has not been investigated. VPA exposure is also regularly associated with lipid accumulation and increased lipid droplet vesicle formation in hepatocytes [118, 120, 150, 153, 184, 218, 219]. Early case reports using skeletal muscle biopsies from VPA-treated patients also showed lipid droplet accumulation between and adjacent to myofibrils [220, 221]. However, whether this was caused by upregulated TAG synthesis or impaired lipolysis was not investigated. In contrast, in a mouse embryonic fibroblast/pre-adipocyte cell line, VPA exposure instead decreased cellular triglyceride levels, suggesting that impaired lipolysis/FA oxidation might be more relevant in causing cellular lipid accumulation [204]. Regardless of cause, if cardiomyocyte steatosis occurs following exposure to either of these drugs, it would likely have an important role in the development of cardiotoxicities, as intracellular FA accumulation has been associated with contractile dysfunction, arrhythmogenesis, and pro-inflammatory signalling [222] (Table 2).

**Table 2:** Summary of evidence for clozapine (CLZ)- and valproate (VPA)-related derangement of fatty acid (FA) metabolism

First author [References]	Experimental model	Exposure	Relevant results
**Supply and cellular uptake**			
*Clozapine*			
Wang [130]	CLZ- or OLZ- taking schizophrenic patients with or without metabolic syndrome	75–900 mg/day (CLZ), 2.5–30 mg/day (OLZ) 0.8–16.8 years	Patients with metabolic syndrome had higher serum FABP4 levels
Pillinger [135]	Systematic review and meta-analysis of 100 randomised controlled trials comparing antipsychotics and placebos in schizophrenic patients	Variable	CLZ treatment increased plasma cholesterol and triglyceride levels
Henderson [132]	CLZ- or risperidone-taking schizophrenic patients	Variable, ≥12 months	Increased plasma triglycerides in CLZ-taking patients
Krakowski [133]	Schizophrenic patients randomised to CLZ vs haloperidol or olanzapine	200–800 mg/day, 12 weeks	Increased plasma cholesterol (vs haloperidol only) and triglyceride levels
Tschoner [137]	Schizophrenic patients assigned to CLZ or olanzapine (pooled) vs other antipsychotics	Not specified, 4 weeks	Increased fasting plasma cholesterol but not triglyceride levels
Kumra [134]	Children and adolescents with schizophrenia, randomised to CLZ or olanzapine treatment	50–700 mg/day (CLZ), 10–30 mg/day (olanzapine), 24 weeks	Increased plasma cholesterol and fasting triglycerides among both treatment groups compared to baseline
Wu [138]	Schizophrenic patients treated with CLZ	200–400 mg/day, 8 weeks	Increased plasma triglyceride levels
Wu [139]	Schizophrenic patients treated with CLZ	200–400 mg/day, 8 weeks	Increased plasma triglycerides and cholesterol levels
Smith [136]	Schizophrenic or schizoaffective patients taking CLZ or other antipsychotics	Mean 416.8 mg/day, ≥ 1 month	Increased fasting plasma triglycerides among CLZ-taking patients
Grover [263]	Diabetic schizophrenic patients treated with CLZ	Not specified, 16 months	Increased triglyceride levels
Liu [141]	Female Sprague–Dawley rats	20 mg/kg/day, 9 weeks	Increased fasting plasma cholesterol levels (≥ 5 weeks)
Jassim [140]	Female Spraque-Dawley rats	25 mg/kg (i.p), Acute	Increased plasma free fatty acids and triglycerides. Decreased plasma cholesterol and phospholipid levels
Wang [149]	ICR mice	12.5/25/50 mg/kg, 6 h or 21 days	Unchanged plasma triglycerides and cholesterol levels
Chen [129]	J774.A1 cells (mouse reticular sarcoma-derived macrophage line)	0.4 µM, 2/18 h	Increased accumulation of oxidised LDL—prevented by cd36 inhibition and siRNA exposure (2 h) Increased CD36 expression
Löffler [131]	SGBS cells (human preadipocyte line)	1.8 µM, 12 days	FABP4 mRNA expression unchanged
*Valproate*			
Phabphal [147]	Fasted epileptic patients treated with VPA vs untreated patients	Not specified, 1–20 years	Unchanged plasma triglyceride and cholesterol levels
Bai [154]	Epileptic patients treated with VPA mono- or polytherapy	Mean 762 mg/day	Development of obesity during treatment associated with single nucleotide polymorphisms in CD36 genes
Tomoum [144]	Children and adolescents with idiopathic epilepsy treated with VPA vs healthy controls	Not specified, 0.6–6.5 years	Decreased plasma cholesterol levels, unchanged triglyceride levels
Beghi [146]	Epileptic patients treated with VPA vs untreated patients	Variable	Increased plasma cholesterol levels
Gatti [155]	Serum samples from healthy controls and type 1 diabetic patients	420 µM, 20 min (at room temperature)	Increased free vs bound fraction of valproic acid in serum from diabetic patients, correlated with free fatty acid concentrations
Thurston [148]	Epileptic children treated with VPA vs other antiepileptics	20–60 mg/kg/day, 0.8–4.5 years	Unchanged plasma free fatty acid levels
Zhang [145]	Male Sprague–Dawley rats	100/500 mg/kg/day (i.g.), 8 weeks	Decreased plasma free fatty acid levels
Ma [151]	Male C57B/6 J mice	500 mg/kg/day (p.o), 30 days	Increased hepatic triglyceride and cholesterol levels. Increased CD36 mRNA expression
	L02 cells (HeLa derivative line)	1/2/5 mM, 72 h	Increased cellular triglyceride (≥ 1 mM) and cholesterol (≥ 2 mM) levels. Increased CD36 mRNA expression
Yan [152]	HepG2 cells (human hepatocellular carcinoma line)	0.5/1/2 mM, 24 or 48 h	Increased cellular triglyceride levels (2 mM for 24 h, ≥1 mM for 48 h). Increased FABP (≥ 0.5 mM) and CD36 (≥ 1 mM, 48 h) mRNA expression
Grünig [153]	HepaRG cells (human hepatocellular carcinoma line)	1/3/6 mM, Acute/3/7 days	Increased mRNA expression of CD36. Decreased mRNA and protein expression of FABP
Chang [150]	FL83B cells (mouse embryonic hepatocyte line)	0.1–10 mM, 24 h	Cellular lipid accumulation unchanged with VPA alone
**Mitochondrial FA uptake**			
*Clozapine*			
Wang [149]	ICR mice	12.5/25/50 mg/kg, 6 h or 21 days	Increased urinary and carnitine levels (≥ 25 mg/kg from 6–24 h, ≥ 12.5 mg/kg from 10–21). Decreased plasma carnitine levels (≥ 25 mg/kg from 6 h – 21 days) Unchanged liver carnitine levels after 6 h, decreased after 21 days (≥ 12.5 mg/kg)
	Isolated proximal tubular cells (from ICR mice) HK-2 cells (human epithelial proximal tubule line) HepG2 cells (human hepatocellular carcinoma line)	1/10/25/50/100 µM, 10 min	Decreased carnitine uptake (≥ 1 µM in proximal tubular, ≥10 µM in HK-2, 100 µM in HepG2 cells)
	MDCK-hOCTN2 cells (canine epithelial kidney cells, transfected with human OCTN2 cDNA)	%1.%2 – 1000 µM, 3 min	Inhibition of carnitine uptake (IC_50_ 1.78 µM)
Kim [169]	Frontal cortex (male Sprague–Dawley rats)	5/10/20 mg/kg (i.p.), acute	Increased ACC (≥ 10 mg/kg) phosphorylation. Increased CPT1c, not CPT1a activity (10 mg/kg)
Kim [168]	C2C12 cells (mouse skeletal muscle myoblast line)	1–9 µM, 1 h	Concentration-dependant increase in ACC phosphorylation
*Valproate*			
Coppola [173]	Epileptic patients treated with VPA vs other antiepileptic drugs	Not specified	Increased risk of hypocarnitinaemia with VPA treatment
Toksoy [175]	Epileptic children treated with VPA vs healthy controls	20 mg/kg/day, 0.5–7 years	Decreased plasma carnitine levels
Beghi [146]	Epileptic patients treated with VPA vs untreated patients	Variable	Higher ratio of plasma acyl- to free carnitine
Melegh [174]	Epileptic children treated with VPA vs healthy controls	Not specified	Decreased plasma carnitine levels
Thurston [172]	Swiss Webster mice	20–30 mg/kg (4-en-VPA, s.c.), Acute or 5 days	Decreased hepatic CoA, acetyl-CoA, and carnitine levels. Increased medium-chain acyl CoA and acyl-to-free carnitine ratio
Thurston [60]	Infant mice, strain not specified	15/60 mg/kg, Acute	Decreased hepatic CoA and acetyl-CoA, free carnitine (≥ 15 mg/kg). Increased plasma acid-soluble CoA esters, acid-insoluble carnitine esters (≥ 15 mg/kg)
Becker [171]	Male Wistar rat	200 mg/kg (i.p.), Acute	Decreased hepatic CoA, acetyl CoA, long-chain acyl-CoA levels. Increased hepatic medium-chain acyl-CoA levels
	Isolated hepatocytes (Female fed Wistar rats)	– 1 mM, 45 min	Decreased acetyl-CoA levels
	Isolated hepatocytes (Fasted male Wistar rats)	1 mM, 60 min	Decreased CoA and acetyl-CoA levels. Increased medium-chain acyl-CoA levels
Wang [176]	Isolated hepatocytes (male Sprague–Dawley rats)	25/50/100/200/500 µM, 24 h	Decreased mRNA expression of CPT1A (≥ 100 µM)
Pirozzi [105]	Rat hepatocytes (male Wistar rats)	1 mM, 24 h	Decreased CPT activity
	HepG2 cells (human hepatocellular carcinoma line)	2 mM, 48 h	Decreased mitochondrial CPT1 expression and activity
Grünig [153]	HepaRG cells (human hepatocellular carcinoma line)	1/3/6 mM, Acute/3/7 days	Increased CPT1 mRNA and protein expression
Kasuya [170]	Isolated hepatic mitochondria (male ddY mice)	0.5–50 mM, 60 min	Unchanged long-chain acyl-CoA synthetase activity, inhibition of medium-chain acyl-CoA synthetase (≥ 10 mM)
**Mitochondrial FA oxidation**			
*Clozapine*			
Albaugh [183]	C57BL6 mice	10 mg/kg (p.o.), Acute	Rapid drop in respiratory exchange ratio (systemic CO_2_ production/O_2_ consumption)
**Valproate**			
Daniels [189]	Isolated working heart preparation (Male Sprague–Dawley)	1 mM, Acute	Decreased FA (16:0 and 8:0) oxidation in the presence of glucose. Increased FA (16:0) oxidation in the presence of pyruvate or lactate without glucose Peak developed left ventricular pressure unchanged except when perfused with lactate or pyruvate instead of glucose
Kesterson [185]	Male Sprague–Dawley rats	700 mg/kg/day (i.p), 5 days	Decreased hepatic CoA and acetyl-CoA levels. Increased hepatic medium-chain CoA, and carnitine levels
Bjorge [188]	Liver homogenate (male Sprague–Dawley rats)	0.2 mM, 15 min	Decreased rate of fatty acid consumption by ~30% and formation of β-oxidation production by ~40%. Exposure to 4-ene VPA decreased the rate of fatty acid consumption by ~40% and the formation of β-oxidation products by ~55%
Silva [186]	Isolated hepatocytes (male Wistar rats)	0.5/1/10 mM, Acute	Decreased fatty acid oxidation rate by 20–80%. Higher potency of inhibition of the oxidation of longer chain lengths (16:0 and 18:1n-9). 4-ene VPA exposure inhibited fatty acid oxidation more potently at all chain lengths
Coudé [85]	Isolated hepatocytes (male Wistar rats)	0.1–5 mM, 60 min	Decreased fatty acid oxidation rate. Decreased cellular acetyl-CoA levels
Turnbull [187]	Isolated hepatocytes (male Wistar rats)	0.1–10 mM, 20–80 min	Decreased fatty acid oxidation
Grünig [153]	HepaRG cells (human hepatocellular carcinoma line)	1/3/6/9/12/21 mM, Acute/3/7 days	Decreased fatty acid oxidation (acute ≥12 mM) recovered by day 3, enhanced by day 7. Unchanged acyl-CoA dehydrogenase activity, decreased β-ketothiolase activity (≥ 12 mM)
Kasuya [170]	Isolated hepatic mitochondria (male ddY mice)	0.5–50 mM, 60 min	Inhibition of medium-and long-chain fatty acid activation and oxidation (50 mM)
**Cellular FA synthesis/accumulation**			
*Clozapine*			
Liu [141]	Female Sprague–Dawley rats	20 mg/kg/day, 9 weeks	Increased hepatic ACC1 mRNA expression
Lauressergues [202]	IHH cells (human immortalised hepatic line)	1/2.5/10/25 µM, 24 h	Increased fatty acid synthase mRNA expression
Kim [169]	Frontal cortex (male Sprague–Dawley rats)	5/10/20 mg/kg (i.p.), acute	Increased ACC (≥ 10 mg/kg) phosphorylation
Fernø [201]	GaMg cells (human glioblastoma line)	30 µM, 1–24 h	Increased expression of fatty acid synthase-encoding genes
Kim [168]	C2C12 cells (mouse skeletal muscle myoblast line)	1–9 µM, 1 h	Concentration-dependant increase in ACC phosphorylation
Chen [216]	Adipose-derived stem cells (Lewis rats)	10 μM, 7 days	Increased ACC gene expression
*Valproate*			
Zhang [145]	Male Sprague–Dawley rats	100/500 mg/kg/day (i.g.), 8 weeks	Decreased plasma and biliary free fatty acid levels
Thurston [60]	Infant mice	15/60 mg/kg, Acute	Decreased hepatic citrate and malate levels (≥ 15 mg/kg)
Wang [176]	Isolated hepatocytes (male Sprague–Dawley rats)	25/50/100/200/500 µM, 24 h	Increased mRNA expression of ACC (≥ 100 µM) and fatty acid synthase (≥ 200 µM)
Becker [171]	Isolated hepatocytes (female fed Wistar rats)	– 1 mM, 45 min	Decreased FA synthesis, citrate levels, and acetyl-CoA
Li [143]	HepaRG cells (human hepatocellular carcinoma line)	346.7 or 693.4 µM, 24 h	Increased cellular triglyceride and FA (16:0) content. Increased mRNA and protein expression of FAS and ACC
Grünig [153]	HepaRG cells (human hepatocellular carcinoma line)	1/3/6 mM, Acute/3/7 days	Increased cellular FFA levels
Yuyama [204]	3T3-L1 cells (lipid-accumulating mouse embryonic fibroblast line)	1 mM, 7 days	Unchanged ACC mRNA expression. Decreased fatty acid synthase mRNA expression
**Triacylglycerol (TAG) synthesis and cellular lipid accumulation**			
*Clozapine*			
Wang [149]	ICR mice	12.5/25/50 mg/kg, 6 h or 21 days	Increased hepatic triglyceride (≥ 25 mg/kg) and cholesterol (≥ 12.5mg/kg) levels
Liu [141]	Female Sprague–Dawley rats	20 mg/kg/day, 9 weeks	Increased hepatic lipid content and lipid droplet size
Jassim [140]	Female Spraque-Dawley rats	25 mg/kg (i.p), Acute	Hepatic lipid accumulation
Vestri [217]	Isolated adipocytes (male Wistar rats)	5–100 μM, 3 h	Increased basal and insulin-stimulated lipogenesis (100 μM). Decreased basal, insulin-induced, and isoproterenol-induced lipolysis
Chen [216]	Adipose-derived stem cells (Lewis rats)	10 μM, 7 days	Increased lipid droplet accumulation
Löffler [131]	SGBS cells (human preadipocyte line)	1.8 µM, 12 days	Triglyceride accumulation unchanged following adipocyte maturation
Lauressergues [202]	IHH cells (human immortalised hepatic line)	1/2.5/10/25 µM, 24 h	Cellular lipid accumulation
Chavarría-Cubel [215]	Huh7 cells (human hepatic carcinoma line)	10 µM, 3/14/16 h	Increased intracellular Dil-LDL content and decreased distance of endolysosomes from nucleus
Fernø [201]	GaMg cells (human glioblastoma line)	30 µM, 1–24 h	Increased cellular cholesterol and triglyceride levels
*Valproate*			
Melegh [220]	Skeletal muscle biopsies from children treated with VPA	Variable	Lipid droplet accumulation between myofibrils, adjacent to mitochondria
	Rats, sex/strain not specified	100 mg/kg/day, 14 days	Hepatic steatosis accompanied by lipid droplet accumulation in skeletal muscle
Gai [120]	Female C57/BJ mice	100 mg/kg/day, 4 weeks	Hepatic lipid accumulation
Tang [118]	Male Sprague–Dawley rats	100 mg/kg/day (i.p.), 5 days	Hepatic microvesicular steatosis
Rodrigues [219]	Human dermal stem cell-derived hepatocytes	27.7 mM, 72 h	Cellular lipid accumulation
Grünig [153]	HepaRG cells (human hepatocellular carcinoma line)	1/3/6 mM, Acute/3/7 days	Cellular lipid accumulation (≥ 3 mM)
Elphick [184]	Huh7 cells (human hepatic carcinoma line)	0.5 mM, 24 h	Lipid droplet accumulation
Chang [150]	FL83B cells (mouse embryonic hepatocyte line)	0.1–10 mM, 24 h	Cellular lipid accumulation unchanged with VPA alone. Concentration-dependent increase in oleate-induced lipid accumulation
Yuyama [204]	3T3-L1 cells (lipid-accumulating mouse embryonic fibroblast line)	1 mM, 7 days	Decreased cellular triglyceride and glycerol levels

### Carbohydrate Metabolism

Cardiomyocytes are supplied with carbohydrates in the form of monosaccharides, such as glucose. Glucose uptake by cardiomyocytes is variable, increasing with metabolic demand during atrial pacing and aerobic exercise [223–225]. Cardiac glucose uptake is also readily influenced by the supply of other substrates, being inversely proportional to plasma FA and ketone levels [226, 227]. Approximately 10–20% of the glucose taken up by the heart is immediately oxidised, though this proportion increases during hyperglycaemia [228, 229]. For this to occur, glucose is first converted to glucose−6-phosphate by hexokinases [230]. This molecule can then undergo glycolysis, involving 10 enzymatic reactions that terminate in the production of pyruvate. The completion of glycolysis results in the nett generation of ATP and NADH [231]. While most steps in glycolysis are reversible, those catalysed by the hexokinases, phosphofructokinase, and pyruvate kinase are essentially irreversible and highly favourable. The activity of these enzymes therefore determines most of the rate of glycolytic flux towards pyruvate production, and they are thus referred to as the glycolytic regulatory enzymes [232]. Their activity is regulated in the short term by product inhibition and allosteric modulation. The hexokinases, for example, have a high affinity for glucose, but are inhibited by the accumulation of the product glucose 6-phosphate [233]. Similarly, high levels of ATP allosterically inhibit phosphofructokinase 1 and pyruvate kinase activity, which are also inhibited by the accumulation of acetyl-CoA and citrate when the TCA cycle is saturated [234–236]. In contrast, when ATP hydrolysis increases, increased AMP concentrations can overcome phosphofructokinase 1 inhibition by ATP [235].

Pyruvate can also be directly taken up by cardiomyocytes through a H^+^/monocarboxylate cotransporter, though typical serum concentrations are lower than those of glucose [237, 238]. During aerobic glycolysis, pyruvate is taken up into the mitochondria through the mitochondrial pyruvate carrier, where it is used to synthesise acetyl-CoA by pyruvate dehydrogenase in a reaction requiring NAD^+^. Conversely, during anaerobic glycolysis, pyruvate is converted to lactate in a reversible reaction catalysed by cytosolic lactate dehydrogenase. This reaction requires NADH, thereby regenerating the NAD^+^ needed for continued glycolytic flux. Lactate is released by the myocardium, with approximately 40–50% being produced from exogenous glucose, increasing to almost 100% during hyperglycaemia [228, 229]. While lactate is generated through anaerobic glycolysis and secreted, the heart is in fact a nett consumer of lactate, with plasma concentrations typically exceeding those of pyruvate [142, 239]. Lactate can also be taken up via a H^+^/monocarboxylate cotransporter and converted to pyruvate by lactate dehydrogenase [240]. Lactate uptake is enhanced with increased serum concentrations at rest and during exercise [210, 225, 241, 242]. Lactate uptake is also directly increased with energy demand in vivo, as has been demonstrated during atrial pacing in humans [223, 243].

CLZ treatment is regularly associated with elevated plasma glucose levels in schizophrenic patients [132–135]. In rats, CLZ administration caused a significant increase in plasma glucose levels within 15 min, which was attributed to an upregulation of hepatic gluconeogenesis [140]. Prolonged exposure to a higher daily dose of CLZ in the same rat strain was reported to transiently decrease fasting plasma glucose levels, though these were increased significantly more following a glucose challenge when compared to control animals [141]. CLZ has also been associated with increased serum pyruvate levels in patients [244]. At the very least, one might predict that CLZ exposure increases cardiomyocyte glucose and pyruvate uptake. However, while CLZ has been shown to acutely increase glucose uptake in a myoblast cell line, it was instead associated with impaired glucose uptake at higher concentrations and a prolonged period of exposure in an embryonic fibroblast cell line [168, 217]. CLZ may also affect carbohydrate oxidation. In a neuronal cell line, CLZ decreased pyruvate dehydrogenase activity when exogenous glucose levels were high, which was associated with a decreased NAD^+^:NADH ratio, but no change in lactate or pyruvate levels [76]. This could indicate that glycolysis is not impaired in these cells, but that the capacity to oxidise NADH is, so that less NAD^+^ is regenerated, thereby decreasing the activity of pyruvate dehydrogenase. This aligns with observations in an endothelial cell line and isolated rat adipocytes that CLZ impairs endogenous respiration, but not glycolysis [75, 217]. This might indicate that CLZ’s effect on carbohydrate oxidation is predominantly mediated by downstream ETS or TCA cycle dysfunction, rather than glucose uptake or glycolysis per se, though to date there has not been any investigation into the drug’s effect in a cardiac experimental model.

VPA inhibits hepatic glycolysis, pyruvate oxidation, and gluconeogenesis, thereby decreasing serum glucose levels and hepatic glucose, pyruvate, and lactate levels in rodent models [60, 83, 85, 148, 171, 185, 187, 245, 246]. However, in fasted patients treated with VPA, plasma glucose levels tend to be unchanged or only modestly decreased [147, 247]. VPA’s effect on glycolysis in other tissues appears to be highly variable. In rat erythrocytes, VPA had no effect on the activity of the glycolytic regulatory enzymes [83]. On the other hand, VPA exposure was modestly inhibitory to glycolysis and respiration in rabbit retina [103]. In mesenchymal stem cells, VPA instead increased glycolytic capacity [95]. By decreasing serum glucose levels, VPA may be expected to decrease cardiac glucose uptake. Whether cardiac glycolysis might also be impaired is unclear. In an isolated working rat heart model, acute VPA exposure shifted substrate utilisation towards glucose oxidation, but away from pyruvate and lactate oxidation. Functionally, VPA exposure only impaired left ventricular pressure development in those hearts perfused without glucose, indicating a reliance on this fuel source [189].

Insulin is an important means of regulating carbohydrate metabolism in the heart [248]. The activation of insulin receptors and insulin-like-growth-factor 1 receptors may stimulate similar signalling pathways in the myocardium [248, 249]. Insulin increases cardiomyocyte glucose uptake by promoting the sarcolemmal translocation and enhancing the activity of the facilitated glucose transporters (GLUT) 1 and 4 [250, 251]. Cardiomyocyte contraction alone, as well as increases in workload, also increase GLUT4 translocation, which may be a relatively more important mechanism for regulating glucose uptake in the heart [252]. This is highlighted by the upregulated GLUT4 content and increased rates of basal glycolysis in cardiac insulin receptor knockout mice, which were comparable to insulin-perfused wild-type hearts. Conversely, in quiescent cardiomyocytes isolated from these mice, glucose uptake was decreased [253]. Insulin also directly regulates glucose metabolism, activating phosphofructokinase−2 to produce fructose 2,6-bisphosphate, which in turn allosterically stimulates phosphofructo-1-kinase, one of the glycolytic regulatory enzymes [254, 255]. It also stimulates glucose oxidation by activating mitochondrial Akt, thereby enhancing pyruvate dehydrogenase activity [256]. Insulin stimulates the sarcolemmal translocation of FAT/CD36 to increase cardiomyocyte FA uptake [257]. Hyperinsulinaemia can drastically increase myocardial glucose uptake and may be able to override the suppressive effects of elevated plasma FAs [258, 259]. Simultaneously, insulin suppresses FA oxidation, partly by reversal of the Randle cycle through its actions in enhancing carbohydrate oxidation (see below), but also by inhibiting adenosine monophosphate-activated kinase activity, leading to increased malonyl-CoA generation by ACC [260, 261]. By increasing FA uptake and suppressing FA oxidation, insulin’s nett effect is likely to direct FAs into synthesis/storage pathways, including myocardial TAG and lipid droplet vesicle formation [262].

CLZ treatment is often associated with the development of type 2 diabetes, whereby systemic glucose control is perturbed by cellular insulin insensitivity, despite elevated plasma insulin concentrations [41, 132, 137–139, 263, 264]. In contrast, sub-acute and acute exposure to CLZ in rats apparently induced hepatic gluconeogenesis and inhibited glycogen synthesis through a decrease in plasma insulin levels [140, 265], whereas at the same daily dose over a longer period of exposure, CLZ impaired glucose tolerance and increased fasting plasma insulin levels in the same rat strain [141]. This aligns with observations in isolated rat adipocytes that acute exposure to supratherapeutic concentrations of CLZ decreased the extent and sensitivity of cellular glucose uptake in response to insulin [217]. In either case, impaired insulin sensitivity or production in diabetes results in hyperglycaemia and elevated plasma FAs. In the liver, this facilitates the upregulation of hepatic ketogenesis and elevated plasma ketone levels, leading to ketoacidosis, of which there exists a plethora of reports in CLZ users [266–280]. CLZ’s association with insulin insensitivity may further result in impaired glucose and FA uptake and lipid storage, despite the elevated serum concentrations described previously, depriving cardiomyocytes of two essential fuel sources. However, this has not been investigated in any cardiac experimental model. VPA, on the other hand, has not been strongly associated with the development of diabetes, changes in plasma insulin levels, or insulin sensitivity [147, 148]. However, several reports of diabetic ketoacidosis in VPA users have been made [276, 281–284]. This is sometimes attributed to the development of acute pancreatitis, causing insulin deficiency rather than insensitivity [285, 286].

FA metabolism is an important regulator of carbohydrate metabolism, via the Randle cycle, through which intermediates of FA and glucose oxidation inhibit the activity of the opposing pathway [287, 288]. For instance, increased circulating levels of FAs decrease the amount of glucose taken up and oxidised by the heart [228]. Cardiac lactate uptake is also inversely proportional to serum FA concentrations [241]. This is facilitated, in part, by enhanced expression of the pyruvate dehydrogenase kinase, which phosphorylates pyruvate dehydrogenase, leading to decreased pyruvate oxidation [289]. Increased pyruvate dehydrogenase kinase activity can also be mediated by the elevated acetyl-CoA concentrations that accompany increased rates of FA oxidation [290]. Elevated concentrations of the TCA cycle intermediate citrate can slow glycolysis by inhibiting phosphofructokinase−1 activity [291]. On the other hand, elevated acetyl-CoA levels can also inhibit the activity of the terminal β-oxidation enzyme, 3-keto-acyl CoA thiolase [292]. Acetyl-CoA might also be exported from the mitochondria via conversion to acyl-carnitine. In the cytosol, the conversion of acetyl-CoA to malonyl-CoA by ACC can then inhibit mitochondrial FA uptake through CPTI [293]. This is important, as any drug-mediated upregulation of the cardiac oxidation of glucose, for example, would similarly downregulate the oxidation of FAs. Similarly, VPA-mediated inhibition of mitochondrial FA oxidation could be expected to cause a shift towards glucose oxidation in cardiomyocytes, as was reported in an isolated working rat heart model, discussed previously [189]. In the presence of CLZ-related downregulation of insulin signalling and impaired cardiomyocyte glucose uptake and glycolysis due to decreased mitochondrial NADH oxidation, the ability of cardiomyocytes to oxidise two major sources of ATP would be impaired (Table 3).

**Table 3:** Summary of evidence for clozapine (CLZ)- and valproate (VPA)-related derangement of carbohydrate metabolism

First author [References]	Experimental model	Exposure	Relevant results
**Supply and cellular uptake**			
*Clozapine*			
Pillinger [135]	Systematic review and meta-analysis of 100 randomised controlled trials	Variable	Increased plasma glucose levels among CLZ-treated patients
Huang [244]	Schizophrenic patients treated with CLZ or olanzapine (non-diabetic) vs healthy controls	Not specified	Higher fasted plasma pyruvate levels
Henderson [132]	CLZ- or risperidone-taking schizophrenic patients	Variable, ≥12 months	Increased fasting plasma glucose levels
Krakowski [133]	Schizophrenic patients randomised to CLZ vs haloperidol or olanzapine	200–800 mg/day, 12 weeks	Increased plasma glucose levels (vs haloperidol and olanzapine)
Kumra [134]	Children and adolescents with schizophrenia, randomised to CLZ or olanzapine treatment	50–700 mg/day (CLZ), 10–30 mg/day (olanzapine), 24 weeks	Increased fasting blood glucose levels among both treatment groups compared to baseline
Liu [141]	Female Sprague–Dawley rats	40 mg/kg/day (p.o.), 9 weeks	Transiently decreased fasting plasma glucose levels. Increased plasma glucose following glucose challenge vs controls
Jassim [140]	Female Spraque-Dawley rats	25 mg/kg (i.p), Acute	Increased plasma glucose levels
Kim [168]	C2C12 cells (mouse skeletal muscle myoblast line)	1–9 µM, 1 h	Increased glucose uptake (≥ 3 µM)
Vestri [217]	3T3-L1 cells (lipid-accumulating mouse embryonic fibroblast line)	1–1000 μM, 3–72 h	Decreased maximal glucose uptake (≥ 5 μM, ≥3 h)
*Valproate*			
Phabphal [147]	Epileptic patients treated with VPA vs untreated patients	Not specified, 1–20 years	Unchanged plasma glucose levels
Turnbull [247]	Fasted human subjects	400 mg (i.v.)/ 1 g (p.o), Acute	Modestly decreased plasma glucose levels
Thurston [148]	Epileptic children treated with VPA vs other antiepileptics	20–60 mg/kg/day, 0.8–4.5 years	Unchanged plasma glucose levels
Tikhonova [83]	Male Wistar rats	100/250/500/1000 mg/kg, Acute	Decreased plasma and erythrocyte glucose levels (≥ 500 mg/kg)
Turnbull [246]	Female BB/E Wistar rats with history of ketoacidosis during insulin therapy	200 mg/kg, acute	Decreased plasma glucose levels
Kesterson [185]	Male Sprague–Dawley rats	700 mg/kg/day (i.p), 5 days	Decreased plasma glucose levels
Thurston [60]	Infant mouse liver and plasma samples	15/60 mg/kg, Acute	Decreased serum glucose levels. Decreased hepatic glucose and glycogen levels (60 mg/kg)
Rogiers [245]	Isolated hepatocytes (male Wistar rats)	0.01–5 mM, 40–90 min	Decreased rate and extent of gluconeogenesis (≥ 0.5 mM)
Turnbull [187]	Isolated hepatocytes (male Wistar rats)	0.1–10 mM, 20–80 min	Decreased gluconeogenesis
Becker [171]	Isolated hepatocytes (male Wistar rats)	1 mM, 60 min	Decreased gluconeogenesis
**Glycolysis (anaerobic/aerobic)**			
*Clozapine*			
Vestri [217]	Isolated adipocytes (male Wistar rats)	5–100 μM, 3 h	Unchanged glucose oxidation (100 μM)
Ota [76]	PC12 cells (rat medullary pheochromocytoma line)	50 µM, 24 h	Unchanged intracellular pyruvate and lactate levels. Decreased lactate levels with high extracellular glucose concentrations, accompanied by decreased NAD^+^ levels, and increased PDH activity
Elmorsy [75]	Human blood brain barrier-derived endothelial cells	~30 µM, 24 h	Unchanged glucose-mediated cellular respiration
*Valproate*			
Daniels [189]	Isolated working heart preparation (Male Sprague–Dawley)	1 mM, Acute	Increased glucose and decreased lactate and pyruvate oxidation in the presence of FA (16:0) Peak developed left ventricular pressure unchanged except when perfused without glucose
Tikhonova [83]	Male Wistar rats	100/250/500/1000 mg/kg, Acute	Unchanged erythrocyte regulatory glycolytic enzyme activity. Decreased NAD^+^/NADH ratio
Coudé [85]	Isolated hepatocytes (male Wistar rats)	0.1–5 mM, 60 min	Decreased cellular lactate/pyruvate ratios
Turnbull [187]	Isolated hepatocytes (male Wistar rats)	0.1–10 mM, 20–80 min	Decreased pyruvate oxidation
Killer [95]	Human mesenchymal stem cells	1 mM, 6 days	Increased rate of glycolysis
Zager [103]	Isolated retina (New Zealand White rabbits)	5 mM, Acute	Slight decrease in rate of glycolysis
**Insulin signalling**			
*Clozapine*			
Grover [263]	Diabetic schizophrenic patients treated with CLZ	Not specified, 16 months	Unchanged HbA1c levels
Ingimarsson [41]	Schizophrenic patients treated with CLZ	Not specified	Increased risk of Type II diabetes
Henderson [132]	CLZ- or risperidone-taking schizophrenic patients	Variable, ≥12 months	Decreased insulin sensitivity
Tschoner [137]	Schizophrenic patients assigned to CLZ or olanzapine vs other antipsychotics	Not specified, 4 weeks	Increased fasting plasma insulin levels and insulin resistance
Wu [139]	Schizophrenic patients treated with CLZ	200–400 mg/day, 8 weeks	Increased incidence of insulin resistance
Wu [138]	Schizophrenic patients treated with CLZ	200–400 mg/day, 8 weeks	Increased plasma insulin levels and incidence of insulin resistance following treatment
Leslie [264]	Schizophrenic patients treated with CLZ vs other antipsychotics	Not specified	Highest risk of diabetes diagnosis and diabetic ketoacidosis
Cao [265]	Male Sprague–Dawley rats	20 mg/kg/day, 4 weeks	Increased fasting plasma glucose level. Decreased plasma insulin level and decreased hepatic PI3K, p-Akt/Akt, p-GSH3β/GSK3β protein expression
Liu [141]	Female Sprague–Dawley rats	20 mg/kg/day, 9 weeks	Increased fasting insulin and impaired glucose tolerance
Jassim [140]	Female Sprague–Dawley rats	25 mg/kg (i.p), Acute	Decreased plasma insulin levels
Vestri [217]	Isolated adipocytes (male Wistar rats)	5–100 μM, 3 h	Decreased insulin-stimulated glucose uptake (≥ 5 μM) and insulin sensitivity (100 μM)
*Valproate*			
Phabphal [147]	Epileptic patients treated with VPA vs untreated patients	Not specified, 1–20 years	Unchanged insulin responsiveness to glucose loading
Thurston [148]	Epileptic children treated with VPA vs other antiepileptics	20–60 mg/kg/day, 0.8–4.5 years	Unchanged plasma insulin levels

### Ketone Metabolism

While the majority of acetyl-CoA produced by cardiac mitochondria comes from the oxidation of FAs or glucose, it can also be supplied by the oxidation of ketone bodies during times of nutrient deprivation or following exercise [294]. Circulating ketone levels vary between 100 and 200 μM but can be as high as 2 mM following carbohydrate restriction, fasting, or prolonged exercise, and may reach levels as high as 10 mM following prolonged starvation [295–298]. Cardiac uptake of ketones tends to be approximately 20 to 30% of their supply, and ketone oxidation contributes to between 3 and 5% of ATP production [299]. Hepatic ketogenesis involves an accumulation of acetyl-CoA when its transformation to citrate is saturated, though the ketogenic amino acids leucine and lysine can be utilised instead [300]. During ketogenesis, two acetyl-CoA molecules are used to produce acetoacetate, which can then be converted to β-hydroxybutyrate, or be spontaneously decarboxylated to form acetone. Ketolysis in the heart involves the conversion of β-hydroxybutyrate to acetoacetate, then acetoacetyl-CoA, before producing acetyl-CoA [301].

CLZ treatment has been associated with an increase in serum ketone levels, outside of the context of diabetic ketoacidosis discussed previously, although whether this relates to a direct effect on hepatic ketogenesis or FA oxidation is unclear [244, 270]. Regardless, elevated ketone concentrations would be expected to increase cardiac uptake and oxidation, though this has not been investigated to date. In contrast, serum VPA levels appear to be inversely proportional to β-hydroxybutyrate levels in patients with epilepsy [148, 172, 175, 247, 302, 303]. Whether VPA-related hypoketonaemia relates to impaired carnitine-mediated FA uptake is contentious, as carnitine supplementation prevents it in isolated rat hepatocytes but not in epileptic patients [174, 304]. In mice and rats, a drop in serum ketone levels has been attributed to impaired hepatic ketogenesis, possibly as a consequence of impaired FA oxidation [60, 85, 171, 172, 185, 246, 305, 306]. In the absence of changes in supply, VPA exposure did not have any acute effect on ketone oxidation in isolated working rat hearts, nor did it have any functional effects in hearts perfused with glucose and ketones [189]. However, decreasing serum ketone levels by VPA administration would likely deprive the heart of an additional, albeit minor, source of ATP. The implications of this become more important when considering VPA’s effect on FA oxidation, so that the reliance of cardiomyocytes on glucose oxidation is further increased.

### Amino Acid Metabolism

Amino acids also make a minor contribution to cardiac ATP production. Their breakdown typically involves transamination and oxidative deamination reactions, producing NH_4_^+^ and a carbon skeleton [307]. NH_4_^+^ is either used in the synthesis of nitrogen-containing compounds, or it enters the urea cycle in the liver [308, 309]. The remaining carbon skeleton can be used in both catabolic and anabolic pathways. Among amino acids, glutamate has been shown to have the greatest cardiac uptake and glutamine the greatest cardiac secretion in patients with atrial fibrillation [142]. This may point to cardiac utilisation of glutamate being shifted away from its conversion to the TCA cycle intermediate α-ketoglutarate and towards its use in the synthesis of glutamine, which allows for the detoxification of NH_4_^+^. Cardiac lactate uptake may also support nitrogen balance through pyruvate’s transamination to form alanine, which was also abundantly secreted by the hearts of these individuals [310]. The branched-chain amino acids (valine, leucine, and isoleucine) were also taken up by the heart in these patients [142]. These can be utilised to produce the TCA cycle intermediate succinyl-CoA as well as well as acetyl-CoA, and the ketone body acetoacetate [311]. Elevated concentrations of TCA cycle intermediates may stimulate cataplerosis. However, in the same group of AF patients, amino acid carbon flux appeared to favour anaplerotic pathways instead. Despite some amino acid uptake, cardiac nitrogen balance was negative in these individuals, indicating nett protein breakdown. Notably, this study was conducted in fasted volunteers, so it is difficult to infer whether the heart remains in negative nitrogen balance regardless of feeding state, as it may cycle through phases of nett protein synthesis and oxidation depending on the availability of other substrates. In a smaller cohort of fasted patients with coronary artery disease, the infusion of branched-chain amino acids increased the rate of protein synthesis to equal that of protein degradation, which was unchanged [312].

Amino acid oxidation also plays an important role in the regulation of hepatic gluconeogenesis via the Cori and Cahill cycles. For instance, peripheral amino acid oxidation causes an increase in pyruvate transamination and thus alanine formation, further stimulating hepatic gluconeogenesis and urea excretion via the Cahill cycle [313]. Similarly, increased peripheral glycolysis, and therefore pyruvate and lactate production, increases hepatic gluconeogenesis via the Cori cycle [314]. The Cori- and Cahill cycles together ensure that under periods of increased peripheral energy demand, and thus increased amino acid and glucose oxidation, there is a similar increase in glucose supply and urea excretion.

While CLZ is not typically associated with altered amino acid metabolism, VPA sometimes is. In humans, VPA administration increases serum levels of lactate, pyruvate, and alanine [247]. In mice, VPA doubled hepatic alanine concentrations [60]. This might be attributable to perturbation of the Cori- and Cahill- cycles through decreased hepatic gluconeogenesis rather than peripheral pyruvate transamination, though neither has been investigated. VPA is often associated with hyperammonaemia in patients [146, 175, 315]. In rats, VPA-induced hyperammonaemia was associated with increased renal uptake of glutamine and glutamate, leading to increased renal ammonia production [304, 316]. This was also attributed to inhibition of urea synthesis in rat hepatocytes, though plasma urea levels apparently increase in rats following VPA exposure [185, 187]. Collectively, this might indicate that there is an increased peripheral amino acid breakdown following VPA exposure, that is not appropriately met with increased hepatic urea excretion. This could be a consequence of perturbations in the oxidation of FAs and production of ketones, so that ATP production becomes more reliant on glucose and amino acid oxidation. Alongside CLZ’s effects on insulin signalling as well as carbohydrate and FA oxidation, the energetic consequences of combined therapy could become severe (Table 4).

**Table 4:** Summary of evidence for clozapine (CLZ)- and valproate (VPA)-related derangement of ketone and amino acid metabolism

First author [References]	Experimental model	Exposure	Relevant results
**Ketone supply/oxidation**			
*Clozapine*			
Huang [244]	Schizophrenic patients treated with CLZ or other antipsychotics (non-diabetic) vs healthy controls	Not specified	Schizophrenic patients had higher plasma β-hydroxybutyrate levels, no difference between antispychotics
Henderson [270]	Schizophrenic patients treated with CLZ or other antipsychotics	Not specified	Highest incidence of diabetic ketoacidosis
*Valproate*			
Tarrant [303]	Epileptic children on a ketogenic diet treated with VPA	4–128 mg/kg	Negative association between plasma VPA and β-hydroxybutyrate levels
Toksoy [175]	Epileptic children treated with VPA vs healthy controls	20 mg/kg/day, 0.5–7 years	Decreased plasma β-hydroxybutyrate levels
Melegh [174]	Epileptic children treated with VPA vs healthy controls	Not specified	Decreased plasma β-hydroxybutyrate levels
Turnbull [247]	Fasted human subjects	400 mg (i.v.)/ 1 g (p.o), Acute	Sharp drop in plasma ketone body levels
Thurston [148]	Epileptic children treated with VPA vs other antiepileptics	20–60 mg/kg/day, 0.8–4.5 years	Lower plasma β-hydroxybutyrate levels
Takeuchi [304]	Isolated hepatocytes (male Wistar rats)	0.1–1 mM, 24 h	Decreased ketone body production
Thurston [172]	Swiss Webster mice	20–30 mg/kg (4-en-VPA, s.c.), 5 days	Decreased plasma β-hydroxybutyrate levels
Turnbull [246]	Female BB/E Wistar rats with history of ketoacidosis during insulin therapy	200 mg/kg, acute	Decreased plasma β-hydroxybutyrate levels
Thurston [60]	Infant mice	15/60 mg/kg, Acute	Decreased plasma ketone levels
Kesterson [185]	Male Sprague–Dawley rats	700 mg/kg/day (i.p), 5 days	Decreased plasma β-hydroxybutyrate levels
Thurston [148]	Mice	100/200 mg/kg/day, 5 days	Decreased plasma β-hydroxybutyrate levels
Olson [305]	Isolated perfused livers (female rats)	0.5 mM, 60 min	Decreased ketone body production
Becker [171]	Male Wistar rat	200 mg/kg (i.p.), Acute	Decreased hepatic β-hydroxybutyrate and ratio of β-hydroxybutyrate to acetoacetate
	Isolated hepatocytes (fasted male Wistar rats)	1 mM, 60 min	Decreased rate of ketone body formation and ratio of β-hydroxybutyrate to acetoacetate
Coudé [85]	Isolated hepatocytes (male Wistar rats)	0.1–5 mM, 60 min	Decreased ketogenesis. Decreased β-hydroxybutyrate/acetoacetate ratio
Daniels [189]	Isolated working heart preparation (Male Sprague–Dawley)	1 mM, Acute	Unchanged ketone oxidation and peak developed left ventricular pressure in the presence of glucose
**Amino acid oxidation and the Cahill cycle**			
*Valproate*			
Toksoy [175]	Epileptic children treated with VPA vs healthy controls	20 mg/kg/day, 0.5–7 years	Decreased plasma carnitine levels
Turnbull [247]	Fasted male subjects	400 mg (i.v.)/ 1 g (p.o), Acute	Increased plasma lactate, pyruvate, and alanine levels
Warter [315]	Fasted male subjects	12 mg/kg/5 min then 2.5 mg/kg/h (i.a.), 6 h	Increased plasma ammonia levels, exacerbated by i.v. or p.o. carbohydrates
Beghi [146]	Epileptic patients treated with VPA vs untreated patients	Variable	Elevated plasma ammonia levels
Thurston [60]	Infant mice	15/60 mg/kg, Acute	Decreased hepatic glutamate and glutamine levels. Increased hepatic pyruvate (≥ 15 mg/kg), lactate (60 mg/kg), α-ketoglutarate, and alanine levels (≥ 15 mg/kg)
Elhamri [316]	Fasted male Wistar rats	200 mg/kg, Acute	Increased renal glutamine and glutamate uptake. Increased renal ammonia release
Kesterson [185]	Male Sprague–Dawley rats	700 mg/kg/day (i.p), 5 days	Increased plasma urea levels
Takeuchi [304]	Isolated hepatocytes (male Wistar rats)	0.1–1 mM, 24 h	Increased ammonia production
Turnbull [187]	Isolated hepatocytes (male Wistar rats)	0.1–10 mM, 20–80 min	Decreased urea synthesis and gluconeogenesis

## Conclusions

This review has provided a summary of the existing evidence for the effects of CLZ and VPA on cellular and systemic energetics, considering specifically how mitochondria and OXPHOS are affected, as well as the oxidation of specific substrates. While this may direct future work, currently no firm conclusions can be made about the role of VPA coadministration and perturbed energetics in the development of CLZ-associated cardiotoxicities. There are many gaps in existing knowledge, including a notable lack of investigation into the combined effects of CLZ and VPA on energetics, with most reporting the effect of either drug in isolation only, making it difficult to infer whether the effects of these drugs are additive or synergistic at the level of the myocardium and/or cardiac mitochondria. To draw firm conclusions surrounding the actions of these drugs together is therefore challenging. However, the most obvious problem is the lack of evidence from cardiac experimental models. In the case of CLZ, the only drug effects to have been confirmed in cardiomyocytes are the impairment of OXPHOS alongside ΔѰ_m_ depolarisation and impaired respiration, as well as the induction of mitochondrial ROS production, with no mechanistic insight into how these changes occur or their functional significance [70–72, 93, 114]. With VPA, the only effects reported in cardiomyocytes were the inhibition of mitochondrial respiration, unchanged mitochondrial ROS production, and a shift in myocardial fuel oxidation away from FAs and toward glucose [93, 123, 189].

Those studies that have been undertaken in cardiac experimental models have not involved consistent or therapeutically relevant concentrations and periods of drug exposure. In the case of CLZ, the therapeutic plasma concentration range is between 1 and 2 μM, and the risk of adverse effects is increased at concentrations above 3 μM (the therapeutic alert level)[77]. Therefore, the use of drug concentrations above 5 μM hardly seems relevant outside the context of drug overdose, yet in vitro work will often exceed this up to 20 times (see Tables 1–4). Similarly, while VPA’s therapeutic plasma concentration range lies between 350 and 700 μM, with a therapeutic alert level of approximately 850 μM, the regular use of concentrations above 2 mM in vitro would be excessive [77]. However, the period of exposure in these works also tends to be much shorter than what would be therapeutically relevant, often in the order of a few hours. It is difficult to infer whether the drug effects reported in these studies would be over- or underestimated. Therefore, it is critically important in future work that the effects of CLZ and VPA not only be determined in a clinically relevant experimental model (e.g. human cardiomyocytes), but that the exposure concentrations and durations used mimic achievable and therapeutically relevant plasma concentrations and exposure periods of these drugs. While therapeutic drug monitoring makes it feasible to determine what relevant extracellular concentrations of CLZ and VPA would be, there is a dearth of evidence regarding their cardiac uptake, and thus clinically relevant intracellular concentrations. Studies of cardiac uptake of both drugs, alone and in combination, would therefore be necessary to inform relevant concentrations of exposure in studies of isolated intracellular organelles such as microsomes and mitochondria.

Another key question that existing evidence does not satisfactorily answer, is how is it possible that VPA would have no cardiac adverse effects alone, while still increasing the risk of CLZ-associated cardiotoxicities? It is possible that there is some interaction between the heart and liver that is relevant in this scenario, through the hepatic metabolism of either drug as well as dietary fuel sources, which would directly influence their cardiac supply as any metabolic products produced by the liver are delivered first to the heart via the vasculature. Therefore, the potential interaction of these organs should not be overlooked.

Developing a greater understanding of the significance of mitochondrial dysfunction and metabolic dysregulation in CLZ-associated cardiotoxicities, and the role of VPA therein, may be an important avenue for future work. In particular, this may inform the identification and validation of clinical biomarkers to facilitate prediction of the risk of cardiac AEs in patients receiving CLZ and VPA, alone or in combination. In order to achieve this, it would be imperative that further work be undertaken to determine the effects of CLZ and VPA on cardiac energetics, to establish their role in the development of cardiotoxicities and identify key cellular targets involved in their pathogenesis. We believe this is a promising avenue for future research to inform the more effective prediction, identification, and management of CLZ-associated cardiotoxicities, facilitating the safer use of this essential medicine.

## Data Availability

No datasets were generated or analysed during the current study.

## Abbreviations

ACC: Acetyl-CoA carboxylase
AE: Adverse effect
ATP: Adenosine triphosphate
CI-IV: Respiratory complexes I-IV
CoA: Coenzyme A
CLZ: Clozapine
CPT: Carnitine palmitoyltransferase
CYP450: Cytochrome P450
ETS: Electron transport system
FA: Fatty acid
FABP: Fatty acid binding protein
FACS: Fatty acyl-CoA synthetase
FADH_2_: Flavin adenine dinucleotide
FAT/CD36: Fatty acid translocase
GLUT: Glucose transporter
mPTP: Mitochondrial permeability transition pore
ΔѰ_m_: Mitochondrial membrane potential
NADH: Nicotinamide adenine dinucleotide
OXPHOS: Oxidative phosphorylation
ROS: Reactive oxygen species
TAG: Triacylglycerol
TCA: Tricarboxylic acid
VPA: Valproic acid
